# O_2_-to-Ar Ratio-Controlled Growth of Ga_2_O_3_ Thin Films by Plasma-Enhanced Thermal Oxidation for Solar-Blind Photodetectors

**DOI:** 10.3390/nano15181397

**Published:** 2025-09-11

**Authors:** Rujun Jiang, Bohan Xiao, Yuna Lu, Zheng Liang, Qijin Cheng

**Affiliations:** School of Electronic Science and Engineering, Xiamen University, Xiamen 361102, China; rujunjiang@stu.xmu.edu.cn (R.J.);

**Keywords:** gallium oxide, plasma-enhanced thermal oxidation, growth mechanism, solar-blind photodetector

## Abstract

Ga_2_O_3_ is an ultra-wide bandgap semiconductor material that has attracted significant attention for deep ultraviolet photodetector applications due to its excellent UV absorption capability and reliable stability. In this study, a novel plasma-enhanced thermal oxidation (PETO) method has been proposed to fabricate Ga_2_O_3_ thin films on the GaN/sapphire substrate in the gas mixture of Ar and O_2_. By adjusting the O_2_-to-Ar ratio (2:1, 4:1, and 8:1), the structural, morphological, and photoelectric properties of the synthesized Ga_2_O_3_ films are systematically studied as a function of the oxidizing atmosphere. It is demonstrated that, at an optimal O_2_-to-Ar ratio of 4:1, the synthesized Ga_2_O_3_ thin film has the largest grain size of 31.4 nm, the fastest growth rate of 427.5 nm/h, as well as the lowest oxygen vacancy concentration of 16.61%. Furthermore, the nucleation and growth of Ga_2_O_3_ thin films on the GaN/sapphire substrate by PETO is proposed. Finally, at the optimized O_2_-to-Ar ratio of 4:1, the metal–semiconductor–metal-structured Ga_2_O_3_-based photodetector achieves a specific detectivity of $2.74\times 10^{13}$ Jones and a solar-blind/visible rejection ratio as high as 116 under a 10 V bias. This work provides a promising approach for the cost-effective fabrication of Ga_2_O_3_ thin films for UV photodetector applications.

## 1. Introduction

Solar-blind ultraviolet (UV) radiation in the 200–280 nm range is strongly absorbed by the stratospheric ozone layer and, therefore, cannot penetrate to the Earth’s surface [1]. As a result, solar-blind photodetectors (SBPDs) are capable of detecting extremely weak signals even under direct sunlight or indoor lighting conditions. Compared to visible and infrared photodetectors, SBPDs offer pronounced advantages such as ultra-low background noise, interference-free signal detection, and robust performance in high-interference environments [2]. These characteristics make them highly valuable for a wide range of applications, including missile warning systems, optical communication, fire detection, and corona discharge monitoring [3,4,5,6]. Among the family of wide bandgap semiconductors, gallium oxide (Ga_2_O_3_) stands out due to its large bandgap of 4.8–5.3 eV, which provides excellent selectivity for solar-blind UV light. In addition, Ga_2_O_3_ exhibits over 80% UV transmittance, along with advantages such as cost effectiveness, high radiation resistance, thermal stability, and chemical durability, which establishes it as a highly promising candidate for deep-UV detection [7,8,9,10,11].

Currently, the main techniques for growing Ga_2_O_3_ thin films include metal-organic chemical vapor deposition (MOCVD), molecular beam epitaxy (MBE), pulsed laser deposition (PLD), halide vapor phase epitaxy, magnetron sputtering, and aerosol-assisted chemical vapor deposition, etc. [12,13]. Among these, MOCVD has a high growth rate suitable for large-scale production, but it involves toxic and expensive metal-organic precursors [14]. Techniques such as MBE and PLD are capable of producing high-quality films, but the growth rate is slow and not suitable for mass production [15,16]. Given the inherent limitations of these traditional techniques, thermal oxidation has emerged in recent years as a promising alternative for the fabrication of Ga_2_O_3_ thin films. This technique involves the oxidation of gallium nitride (GaN) into Ga_2_O_3_ in an oxygen-rich atmosphere. It is noteworthy that changing material composition (in this case, from GaN to Ga_2_O_3_) via high-temperature elemental substitution is a widely used approach in various material systems, particularly in 2D materials with large surface-to-volume ratios [17]. Most previous reports on Ga_2_O_3_-based photodetectors prepared via thermal oxidation are focused on Ga_2_O_3_ nanowires [18,19,20,21,22] or Ga_2_O_3_-GaN core–shell nanostructures [23,24,25], rather than crystalline Ga_2_O_3_ thin films. Moreover, thermally oxidized Ga_2_O_3_ often contains residual GaN phase, which is detrimental for solar-blind ultraviolet detection due to GaN’s photoresponse near 365 nm, thereby compromising the UV–visible rejection ratio of the device [26].

In our previous study, β-Ga_2_O_3_ thin films were successfully fabricated on GaN/sapphire substrates using a novel plasma-enhanced thermal oxidation approach. These films showed a rapid growth rate (~409.9 nm/h), a smooth surface with low root-mean-square roughness, and a distinct preferred orientation along the ($\bar{2}01$) crystal plane [27]. In particular, compared with the thermal oxidation method, this approach offers advantages such as a faster growth rate, reduced residual GaN grains, and improved crystallinity [27]. Furthermore, the Ga_2_O_3_ thin films fabricated using this method have demonstrated promising performance in solar-blind ultraviolet photodetector applications. During the process of PETO, the composition of the oxidizing atmosphere is a critical factor influencing the film’s structural quality and surface properties. Therefore, adjusting the oxygen-to-inert gas (e.g., argon) ratio in the oxidation environment is considered as a key strategy for controlling the oxidation rate, suppressing defect formation, and improving crystal quality. This study systematically investigates the effect of varying O_2_-to-Ar ratios on the structural evolution, surface morphology, and photoelectric performance of Ga_2_O_3_ films formed via PETO. The goal is to establish a theoretical foundation and provide technical guidance for optimizing the fabrication process of Ga_2_O_3_ thin films and enhancing the performance of SBPDs.

## 2. Experimental Section

### 2.1. Growth of Ga_2_O_3_ Thin Films

**Figure 1:**
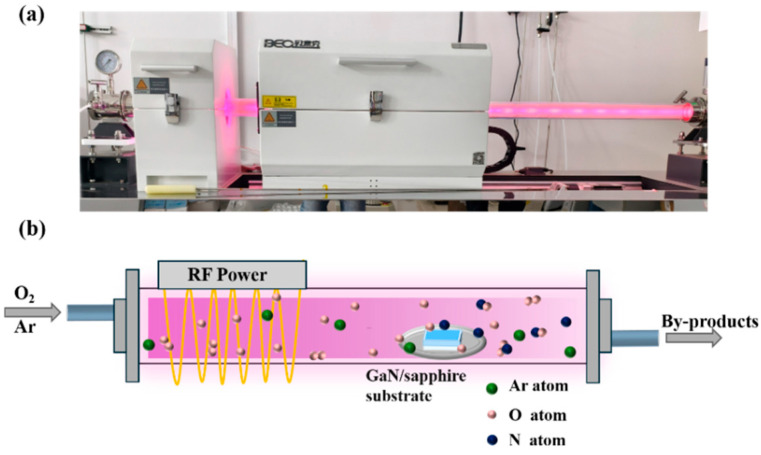
(**a**) Photograph and (**b**) schematic illustration of the RF plasma-enhanced horizontal tube furnace deposition system operating under the glow discharge of an oxygen and argon gas mixture.

An undoped GaN thin film grown on a c-plane sapphire substrate by MOCVD from Hefei Kejing Co., Ltd., located in Hefei, China, was selected as the starting material for the experiment. To remove native oxides and surface contaminants, the GaN/sapphire substrate was ultrasonically cleaned in acetone, ethanol, and deionized water for 15 min in each solvent. The cleaned GaN/sapphire substrate was placed at the center of a quartz boat and subsequently positioned at the center of the heating zone in the radiofrequency (RF) plasma-enhanced horizontal tube furnace deposition system (Anhui BEQ Equipment Technology Co., Ltd., located in Hefei, China; Type: BTF-1200C-PECVD-500A). Thereafter, the mechanical pump was turned on to evacuate the tube furnace to a pressure below 10 Pa, after which the heating program was initiated to raise the temperature to 900 °C. Once the desired temperature was reached, the gas flow control system was activated to introduce oxygen and argon at specific flow rates (80 sccm for oxygen, and 10, 20, or 40 sccm for argon, corresponding to O_2_-to-Ar ratios of 8:1, 4:1, and 2:1, respectively). The total working pressure was maintained at 220 Pa for all three gas ratio conditions. During each experiment, an RF power of 300 W was applied to ionize the gas, and a stable glow discharge was observed. Plasma-enhanced thermal oxidation was carried out for 1 h under constant temperature and gas flow conditions. After oxidation, the RF power supply and gas flow were turned off, and the tube furnace pressure was kept below 10 Pa during natural cooling to room temperature. Once cooled, the vacuum pump was switched off, the tube furnace was opened, and the sample was removed. Figure 1a,b present a photograph and a schematic illustration of the RF plasma-enhanced horizontal tube furnace deposition system operating under the glow discharge of an oxygen and argon gas mixture, respectively.

### 2.2. Photodetector Fabrication

**Figure 2:**
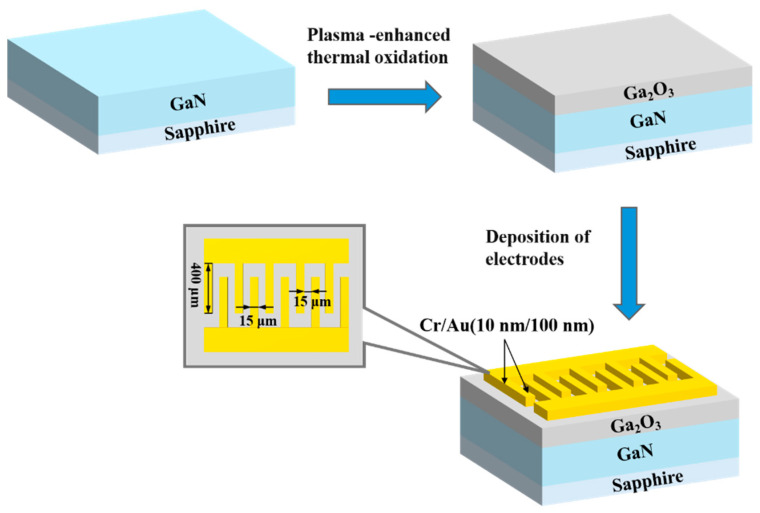
Process flow diagram for manufacturing Ga_2_O_3_-based photodetectors. The inset shows the detailed interdigitated electrode pattern.

The deposited gallium oxide film was utilized to fabricate Ga_2_O_3_/GaN photodetectors. A layer of UV-positive photoresist (AZ 5214) was spin-coated onto the grown Ga_2_O_3_ film, followed by soft baking at 96 °C for 4 min to enhance adhesion. The electrode mask, designed in a metal–semiconductor–metal (MSM) configuration, was then aligned with the substrate and exposed to ultraviolet light for 15 s. After exposure, the sample was developed to remove the unexposed photoresist. Subsequently, Cr/Au (10 nm/100 nm) electrodes were deposited via electron beam evaporation. The remaining photoresist was lifted off to complete the fabrication of Cr/Au metal electrodes. The interdigitated electrode pattern features a finger length of 400 μm, a finger width of 15 μm, and a finger spacing of 15 μm. The overall fabrication process of the Ga_2_O_3_-based photodetector is depicted in Figure 2.

### 2.3. Material and Device Characterization

During the Ga_2_O_3_ film growth, the evolution of plasma species was continuously monitored in real time using optical emission spectroscopy (OES, Avantes ULS2048 from Avantes China, located in Beijing, China). The crystallization behavior of the oxidized samples was investigated with an XRD-7000 diffractometer (Shimadzu, located in Kyoto, Japan) employing Cu Kα ray (wavelength λ = 1.5405 Å). The surface morphology and cross-section of Ga_2_O_3_ thin films were measured using field emission scanning electron microscope (SEM, ZEISS Sigma 300 from Carl Zeiss AG, located in Oberkochen, Germany). Narrow-scan spectra of Ga 2*p*_3/2_ and O 1*s* were recorded using X-ray photoelectron spectroscopy (XPS, Thermo Kalpha ESCALAB 250XI from Thermofish Scientific, located in Shanghai, China) with Al Kα X-ray (hν = 1486.6 eV). The photoelectric test system mainly consisted of a Keithley 2450 digital source meter, a xenon lamp (200–2500 nm), a 300 mm focal length monochromator (spectral resolution 0.1 nm @ 435.84 nm @ 1200 g/mm, wavelength accuracy ± 0.2 nm, wavelength repeatability ± 0.1 nm), an oscilloscope (Siglnent SDS 2102 from Siglnent Technologies, located in Shenzhen, China), a dark box, a probe station, a computer, and other components. Based on this system, the photoelectric characteristics of the fabricated photodetector, such as current–voltage (I–V) characteristic, current–time (I–t) characteristic, and responsivity (*R*), were tested. The photocurrent and responsivity of the fabricated photodetector were calibrated and measured using a standard silicon photodetector.

## 3. Results and Discussion

**Figure 3:**
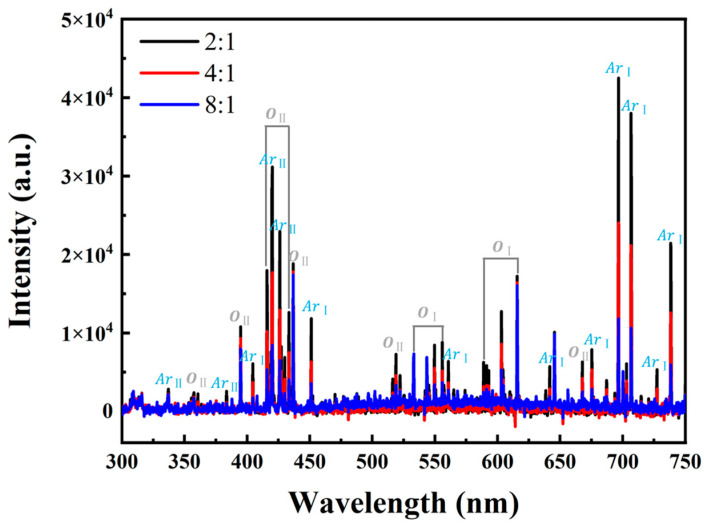
OES spectra under different O_2_-to-Ar ratios.

To elucidate the variation of plasma characteristics under different O_2_-to-Ar ratios, the plasma components during the glow discharge were monitored in real time using OES. The corresponding results are illustrated in Figure 3, which clearly demonstrates the presence of a series of characteristic peaks associated with oxygen atoms (*O*_Ⅰ_), singly ionized oxygen ions (*O*_II_), argon atoms (*Ar*_Ⅰ_), and singly ionized argon ions (*Ar*_II_). Specifically, the most intense emission lines of *O*_Ⅰ_ and *O*_II_ appear at 615 and 437 nm, respectively, whereas those of *Ar*_Ⅰ_ and *Ar*_II_ are observed at 697 and 420 nm, respectively. As the proportion of argon in the gas mixture of argon and oxygen increases, the intensities of the oxygen-related spectral lines are markedly enhanced, thereby suggesting a significant improvement in the activity of oxygen species.

**Figure 4:**
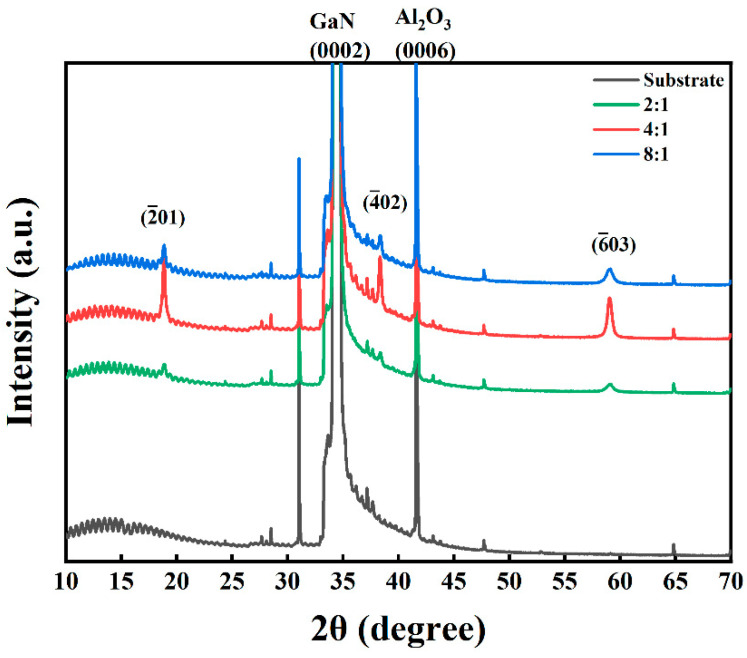
XRD patterns of the synthesized Ga_2_O_3_ thin films with different O_2_-to-Ar ratios.

Figure 4 presents the XRD patterns of Ga_2_O_3_ thin films synthesized by PETO on the GaN/sapphire substrate. The diffraction pattern of the GaN/sapphire substrate reveals two distinct peaks at 34.51°and 41.68°, corresponding to the (0002) crystal plane of GaN (JCPDS No.74-0243) and the (0006) crystal plane of Al_2_O_3_ (JCPDS No.71-1123), respectively. This confirms that the GaN epitaxial layer grown on the c-plane sapphire substrate via MOCVD is oriented along the (0001) crystal plane, with the epitaxial relationship GaN (0001) || Al_2_O_3_ (0001). In the oxidized samples, characteristic diffraction peaks are observed at 18.88°, 38.26°, and 58.9°, corresponding to the ($\bar{2}01$), ($\bar{4}02$), and ($\bar{6}03$) crystal planes of β-Ga_2_O_3_ (JCPDS No.00-043-1012) [28], respectively. No additional diffraction peaks from other crystal planes are detected, indicating that β-Ga_2_O_3_ thin films with a preferential {$\bar{2}01$} family of planes can be successfully grown under plasma-enhanced thermal oxidation with varying O_2_-to-Ar ratios.

The XRD peak corresponding to the ($\bar{2}01$) crystal plane of the oxidized samples was fitted, and the grain size of the Ga_2_O_3_ film was calculated using the following Debye-Scherrer formula:

(1) $$d=\frac{0.9\lambda }{B\cos\theta }$$

where *d* is the average crystallite size of Ga_2_O_3_, *λ* indicates the incident X-ray wavelength, *B* is the peak’s full width at half maximum, and *θ* is the Bragg diffraction angle [29]. The calculated grain sizes of the synthesized Ga_2_O_3_ thin films at O_2_-to-Ar ratios of 8:1, 4:1, and 2:1 are 26.3, 31.4, and 9.4 nm, respectively. It is evident that the grain size is the largest at an O_2_-to-Ar ratio of 4:1, while the grain size is the smallest at an O_2_-to-Ar ratio of 2:1. Notably, the total gas flow rate in the tube furnace is the highest for the oxidized sample synthesized at an O_2_-to-Ar ratio of 2:1 (120 sccm), which may influence the formation of gallium oxide nuclei and, consequently, affect the grain size of the synthesized Ga_2_O_3_ film. Based on the measured XRD patterns, the highest characteristic peak intensity of β-Ga_2_O_3_ is observed at the O_2_-to-Ar ratio of 4:1, suggesting that the crystal quality is best under this condition.

**Figure 5:**
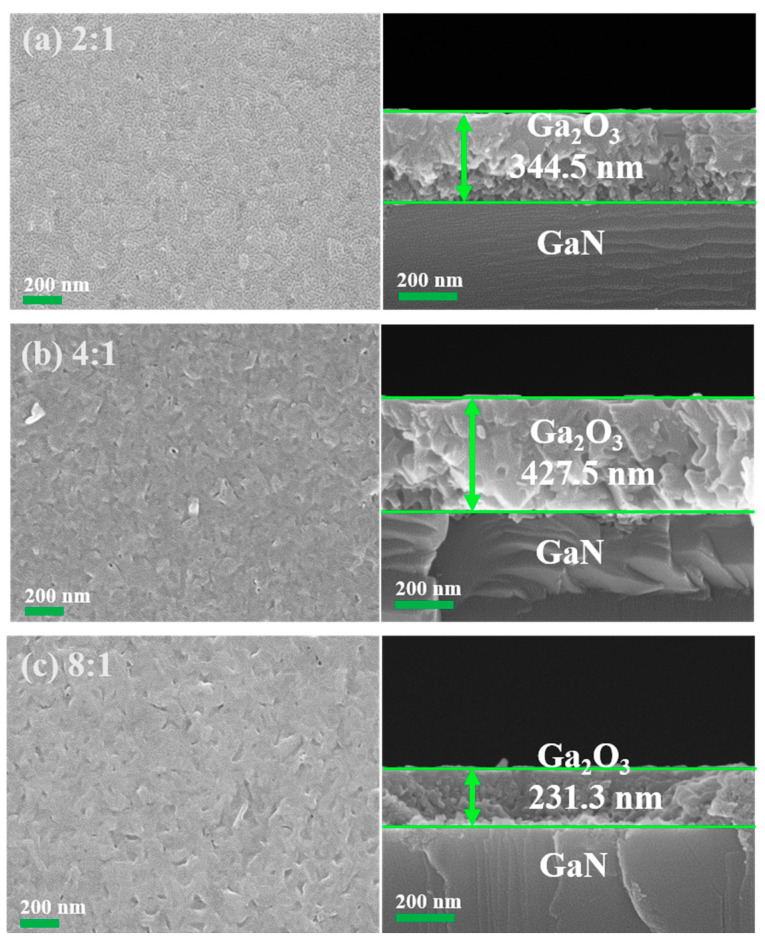
Typical surface morphologies and corresponding cross-sections of the synthesized Ga_2_O_3_ thin films with different O_2_-to-Ar ratios of (**a**) 2:1, (**b**) 4:1, and (**c**) 8:1, respectively.

The morphology and microstructure of the oxidized samples were analyzed using SEM. Figure 5a–c show the surface morphology and cross-section of the synthesized Ga_2_O_3_ thin films with different O_2_-to-Ar ratios. As shown on the left side of Figure 5a, when the O_2_-to-Ar ratio is 2:1, a flat film surface with uniformly distributed nanoparticles is observed. In contrast, when the O_2_-to-Ar ratio is 4:1, slightly irregular shapes and nanoparticles begin to appear on the surface, as shown on the left side of Figure 5b. As the O_2_-to-Ar ratio increases, small nanoparticles gradually transform into large nanoparticles. During the grain merging process, the surface becomes rough, which suggests that the increase in the O_2_-to-Ar ratio may influence the nucleation and growth of the film. With a further increase of O_2_-to-Ar ratio to 8:1, the surface roughness further increases, as shown on the left side of Figure 5c. All samples exhibit a polycrystalline surface with similar surface morphology.

To determine the film thickness, cross-sections of the oxidized samples were examined using SEM. The thicknesses of the synthesized Ga_2_O_3_ thin films using PETO with O_2_-to-Ar ratios of 2:1, 4:1, and 8:1 were measured to be 344.5, 427.5, and 231.3 nm, respectively, as shown on the right side of Figure 5a–c. Among these, the oxidized sample with an O_2_-to-Ar ratio of 4:1 exhibits the largest thickness, indicating that the film growth rate is the highest under these conditions. Conversely, the oxidized sample with an O_2_-to-Ar ratio of 8:1 has the lowest thickness. This may be due to the relatively low argon flow rate in this case, which makes it difficult to effectively produce the plasma. As a result, the ionization degree is low, and a small number of chemically active atomic oxygen-based radicals reduce the overall oxidation rate [27].

**Figure 6:**
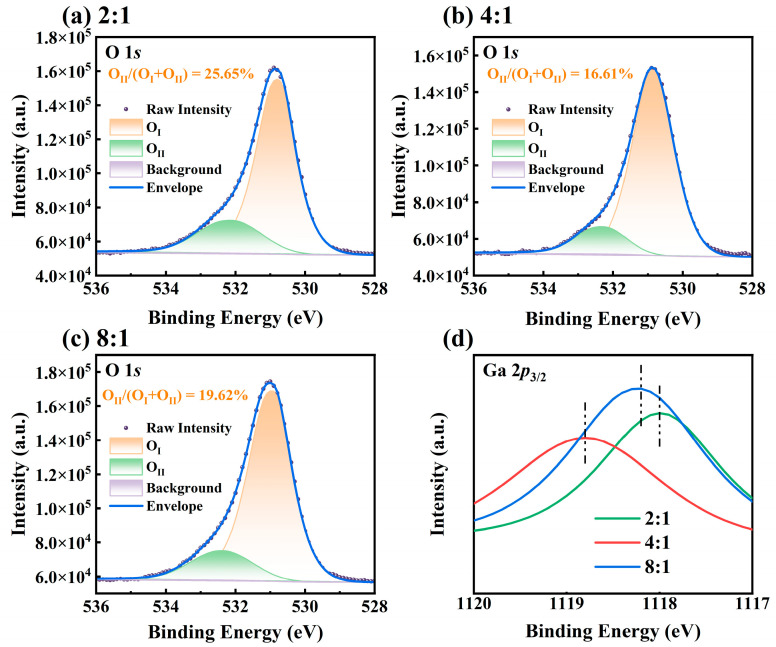
XPS characterization of Ga_2_O_3_ thin films prepared under different O_2_-to-Ar ratios, including narrow-scan O 1*s* spectra with peak fitting at ratios of (**a**) 2:1, (**b**) 4:1, and (**c**) 8:1 and (**d**) the corresponding narrow-scan Ga 2*p*_3/2_ spectra.

XPS was employed to examine the chemical states of Ga_2_O_3_ films and determine the concentration of oxygen vacancy defects. Figure 6a–c describe the narrow-scan O 1*s* XPS spectra of Ga_2_O_3_ thin films prepared with different O_2_-to-Ar ratios. The binding energy was calibrated using the C 1*s* peak at 284.8 eV [30]. It can be observed that the O 1*s* peak exhibits a noticeable asymmetry. After Gaussian fitting, each O 1*s* spectrum can be decomposed into two components: *O*_I_ and *O*_II_. *O*_Ⅰ_, at a lower binding energy of approximately 530.7 eV, which corresponds to the O–-Ga chemical bond in the β-Ga_2_O_3_ film, while the higher binding energy component, *O*_II_, at about 532.1 eV, is generally attributed to the *O*^2−^ ions in oxygen-deficient regions [31]. The relative concentration of each component is determined from the corresponding peak area, and the oxygen vacancy concentration is estimated using the area ratio *O*_II_/*O*_Ⅰ_ + *O*_II_). The calculated oxygen vacancy concentrations of the synthesized Ga_2_O_3_ thin films with O_2_-to-Ar ratios of 2:1, 4:1, and 8:1 are 25.65%, 16.61%, and 19.62%, respectively. It is well known that argon gas is more easily ionized, generating high-energy ${Ar}^{+}$ ions. The produced plasma with a high argon flow rate may bombard the film surface, causing structural defects such as oxygen vacancies [32]. This explains why the oxidized sample with an O_2_-to-Ar ratio of 2:1 has the highest oxygen vacancy concentration.

Figure 6d presents the narrow-scan Ga 2*p*_3/2_ spectra of Ga_2_O_3_ films prepared under different O_2_-to-Ar ratios. The highest binding energy of 1118.8 eV is observed at an O_2_-to-Ar ratio of 4:1, whereas the lowest binding energy of 1118.0 eV occurs at an O_2_-to-Ar ratio of 2:1. Under different oxidation conditions, the variation trend of the binding energy of Ga 2*p*_3/2_ is opposite to that of the oxygen vacancy concentration. Oxygen vacancies act as donor impurities in gallium oxide, providing free electrons, which increases the electron density of nearby Ga atoms and, thus, reduces the binding energy of Ga 2*p*_3/2_ (i.e., it is easier to strip electrons) [33].

**Figure 7:**
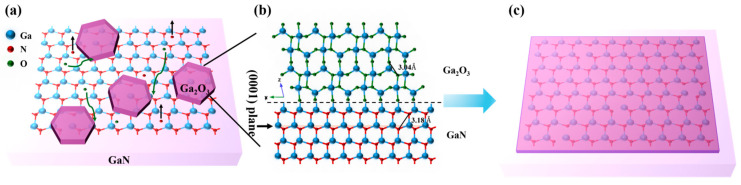
Schematic diagram of the nucleation and growth of Ga_2_O_3_ thin films on the GaN/sapphire substrate by plasma-enhanced thermal oxidation: (**a**) Island-like growth of Ga_2_O_3_; (**b**) Bond formation between Ga_2_O_3_ and GaN; (**c**) The island structure coalesces to form a continuous film.

Now, let us briefly discuss the growth mechanism of Ga_2_O_3_ thin films formed by PETO on the GaN/sapphire substrate. Figure 7 illustrates the schematic diagram of the nucleation and growth of Ga_2_O_3_ thin films on the GaN/sapphire substrate. Under the influence of the electric field existing in the plasma, oxygen molecules are effectively dissociated to form a large number of highly chemically active atomic oxygen-based radicals. The abundance of these atomic oxygen-based radicals in the plasma environment significantly accelerates the growth rate of the Ga_2_O_3_ film [34]. Moreover, these atomic oxygen-based radicals exhibit strong electrophilicity and tend to adsorb onto the GaN surface, preferentially binding to high-energy sites such as steps, dislocations, and other crystal defects, where the chemical reactivity is significantly enhanced. At the same time, Ga–N bonds within the GaN lattice begin to break, and nitrogen is released in the form of NO*_x_* gas, resulting in a Ga-rich surface with under-coordinated Ga atoms or clusters. These exposed Ga atoms subsequently react with the adsorbed atomic oxygen-based radicals to form Ga–O bonds, leading to the gradual formation of Ga_2_O_3_. In the initial stages, Ga_2_O_3_ tends to nucleate on the GaN surface in the form of isolated islands, following a Volmer–Weber growth mode. This growth behavior is attributed to the significant lattice mismatch between Ga_2_O_3_ and GaN, which favors the formation of discrete oxide patches rather than a layer-by-layer growth [35]. As the oxidation reaction proceeds, these islands grow, merge, and eventually coalesce into a continuous Ga_2_O_3_ thin film. Argon, as an inert gas, does not chemically react with GaN in the plasma environment. However, through physical bombardment, it contributes to an increase in the electron temperature. The elevated electron temperature promotes the dissociation of oxygen molecules, generating more reactive atomic oxygen-based radicals (as shown in Figure 3) and thereby accelerating the oxidation reaction. Simultaneously, momentum transfer resulting from Ar collisions leads to the preferential sputtering of nitrogen atoms from the GaN surface, exposing gallium atoms for subsequent oxidation [32]. It is noteworthy that the proposed mechanism, i.e., initial nucleation and growth of the new composition begins at defect sites, is also reported in 2D materials, showcasing similar mechanisms at play in both GaN and 2D materials [36].

As shown in Figure 7b, although β-Ga_2_O_3_ belongs to the monoclinic crystal system, the arrangement of oxygen atoms on the β-Ga_2_O_3_ ($\bar{2}01$) crystal plane is quasi-hexagonal, similar to the arrangement of nitrogen atoms on the GaN (0001) crystal plane. The adjacent N-N atomic spacing on the GaN (0001) crystal plane is 0.318 nm, whereas the adjacent O-O atomic spacing on the β-Ga_2_O_3_ ($\bar{2}01$) crystal plane is 0.304 nm, which is 4.4% shorter than that of GaN [37]. Simultaneously, it can be observed in Figure 4 that β-Ga_2_O_3_ thin films prepared with PETO exhibit a distinct preferential orientation. Based on this, it can be concluded that the growth orientation of β-Ga_2_O_3_ thin films fabricated with plasma-enhanced thermal oxidation on the GaN/sapphire substrate follows β-Ga_2_O_3_ ($\bar{2}01$) || GaN (0001), which is consistent with the results obtained from MOCVD epitaxy [38,39] and conventional thermal oxidation [22,40,41,42,43].

**Figure 8:**
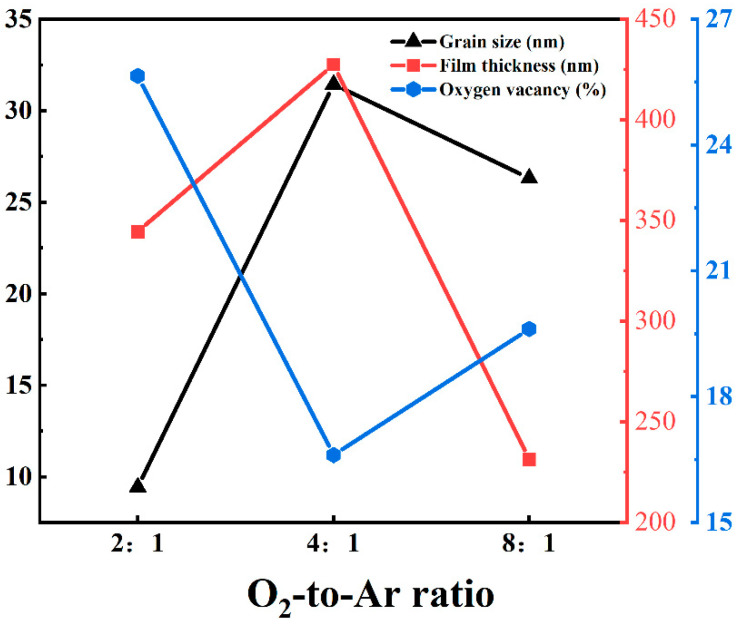
Comparison of grain size, thickness, and oxygen vacancy concentration of β-Ga_2_O_3_ films grown under different O_2_-to-Ar ratios.

Considering the grain size, film thickness, and oxygen vacancy concentration, as shown in Figure 8, the β-Ga_2_O_3_ film prepared with an O_2_-to-Ar ratio of 4:1 exhibits better quality. Therefore, the β-Ga_2_O_3_ film grown under this condition was selected for the fabrication of a SBPD.

**Figure 9:**
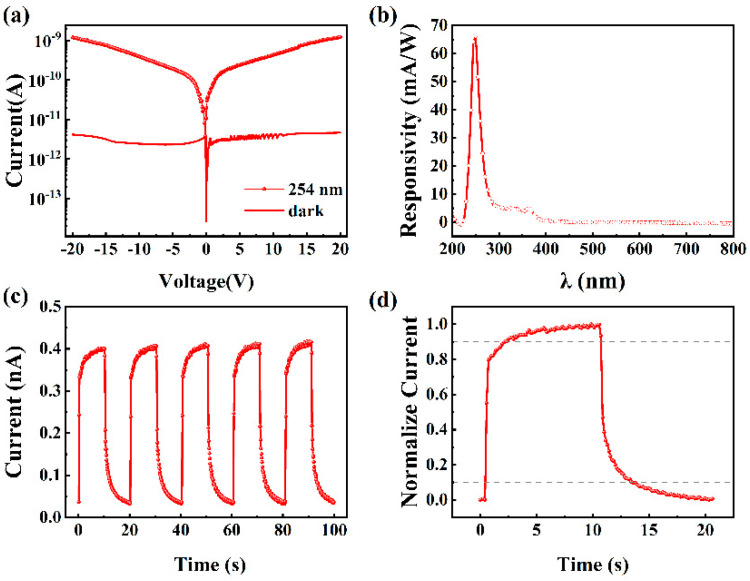
(**a**) I–V characteristics of the fabricated photodetector under dark state and 254 nm illumination, (**b**) spectral responsivity, (**c**) multi-cycle I–t characteristics, and (**d**) normalized single-cycle I–t characteristic curve of the fabricated photodetector under 254 nm illumination at a bias of 10 V.

The oxidized sample grown with an O_2_-to-Ar ratio of 4:1 was fabricated into an MSM-type photodetector, and its current–voltage characteristics under dark and 254 nm illumination are shown in Figure 9a. At a bias of 10 V, the dark current of the device is $3.75\times 10^{-12}$ A, and the photocurrent is $4.29\times 10^{-10}$ A. The sensitivity of a photodetector to the incident light at a particular wavelength can be quantified by the photo-to-dark current ratio (*PDCR*), which is calculated as follows [44]:

(2) $$PDCR=\frac{I_{photo}-I_{dark}}{I_{dark}}$$

where $I_{photo}$ is the current generated when the photodetector is illuminated by the incident light of a specific wavelength, and $I_{dark}$ is the current generated under dark conditions. The calculated *PDCR* at a bias of 10 V is 114 in this work.

Responsivity is defined as the photocurrent generated by the photodetector per unit of incident light power, with a unit of A/W. It is used to measure the ability of a photodetector to convert optical signals into electrical signals, and its expression is [45]

(3) $$R=\frac{I_{photo}-I_{dark}}{P_{\lambda }S}$$

where *R* is the responsivity, $P_{\lambda }$ is the light power density (usually expressed in μW/cm^2^) of the incident light with a wavelength of *λ*, and *S* is the effective area of the photodetector illuminated by light (*S* is 0.226 mm^2^ in this work). Figure 9b shows the spectral responsivity of the fabricated photodetector at a bias of 10 V. The peak responsivity is located near 250 nm, corresponding to the absorption edge of β-Ga_2_O_3_, with a corresponding responsivity value of 66.2 mA/W. Due to the absorption of near-ultraviolet light (280–380 nm) by GaN, the prepared photodetector shows a slight response at 365 nm. Here, we use the ratio of the responsivity at 250 nm to 365 nm or 400 nm to represent the spectral selectivity of the fabricated photodetector. It is calculated that the device’s solar-blind/UV light rejection ratio, defined as $R_{250nm}/R_{365nm}$, reaches 13.79, while its solar-blind/visible light rejection ratio, defined as $R_{250nm}/R_{400nm}$, is 116.

Detectivity (*D*) is one of the key performance indicators of a photodetector, representing its ability to effectively detect light signals under specific lighting conditions. The higher the detectivity is, the more efficiently the photodetector can detect the optical signal at a given optical power and noise level. To evaluate the detectivity of different photodetectors and eliminate the influence of differences in area, shape, and bandwidth, the detectivity is normalized to obtain the specific detectivity (D*). D* is expressed as follows [46,47]:

(4) $$D^{*}=\frac{R\sqrt{S}}{\sqrt{2qI_{dark}}}$$

where *q* is the charge of an electron. The specific detectivity of the fabricated photodetector is calculated to be 2.74 × 10^13^ Jones.

The current–time characteristics of the fabricated photodetector were measured under 254 nm illumination at a bias of 10 V, where the light was turned on and off every 10 s. As shown in Figure 9c, when the UV light is turned on, the current increases slowly, and when the UV light is turned off, the current decreases gradually, exhibiting a clear persistent photoconductivity effect. This behavior is attributed to the presence of oxygen vacancy-related carrier traps in the gallium oxide film, which capture photogenerated carriers. The slow release of these carriers leads to a prolonged response time [6]. Over multiple cycles, the device demonstrates a relatively stable I–t response with high repeatability. The response time describes how quickly a photodetector responds to a light signal, encompassing both rise time and fall time. The rise time ($\tau_{r}$) signifies how long it takes for the current to grow from 10% to 90% of its peak value, and the fall time ($\tau_{d}$) signifies the time for the current to decline from 90% to 10% of its peak value. To obtain the response time of the fabricated photodetector, the stable single-cycle normalized current–time characteristic curve from its I–t characteristics is plotted, as shown in Figure 9d. The obtained $\tau_{r}$ and $\tau_{d}$ of the device are 1.73 and 2.78 s, respectively. The slow rise and fall times obtained in this work are attributed to the high density of oxygen vacancies in the fabricated Ga_2_O_3_ thin film. In the future, we will focus on further reducing the oxygen vacancy concentration by more finely tuning the O_2_/Ar ratio (e.g., 5:1, 6:1, 7:1), adjusting the RF power as well as the growth temperature and applying post-annealing in the oxygen ambient, etc.

**Figure 10:**
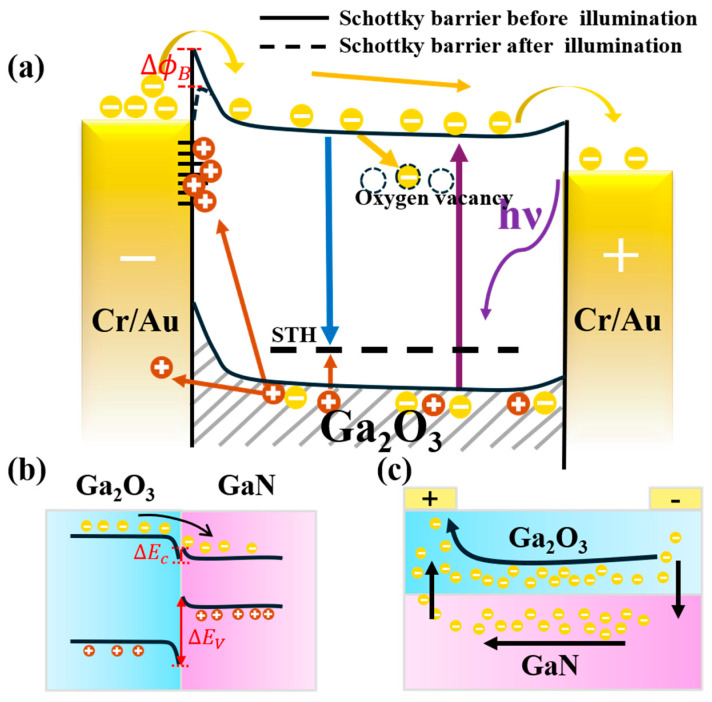
(**a**) Schematic energy band diagram of an MSM-structured Ga_2_O_3_-based photodetector under UV illumination and applied bias; (**b**) band alignment diagram of the heterojunction formed between Ga_2_O_3_ and GaN; (**c**) schematic illustration of electron transport pathways in the heterojunction formed between Ga_2_O_3_ and GaN.

To better understand the influence of oxygen vacancies on the performance of Ga_2_O_3_-based SBPDs, Figure 10a illustrates the schematic energy band diagram of an MSM-structured Ga_2_O_3_-based photodetector under UV illumination and applied bias. In the photodetector, the photoresponse is a complex process involving the generation, capturing, and recombination of electron–hole pairs [48]. Under UV illumination, photogenerated carriers are created in Ga_2_O_3_, where electrons drift toward the positive electrode and holes drift toward the negative electrode. Due to the inherently low mobility of holes in Ga_2_O_3_, holes are readily captured by defect states, forming self-trapped holes (STH) [49]. These trapped holes can subsequently recombine with conduction band electrons, thereby reducing the collection efficiency of photogenerated carriers. Oxygen vacancies are common intrinsic defects in Ga_2_O_3_. Under ultraviolet illumination, they typically exist in positively charged states (such as $V_{O}^{+}$ or $V_{O}^{2+}$), which can attract and capture photo-generated electrons, forming electron traps [50]. This process enhances non-radiative recombination of electrons and may also induce persistent photoconductivity, resulting in increased dark current and response lag in the device. On the other hand, under reverse bias operation, photo-generated holes are driven toward the metal/Ga_2_O_3_ interface by the built-in electric field. At this interface, numerous interface states are typically present, which can act as effective hole trapping centers [46]. When minority carrier holes are captured by these interface states, the spatial charge distribution near the junction is altered, weakening the built-in electric field and reducing the Schottky barrier height. As a result, the barrier’s ability to block electron injection is diminished, leading to an increased current under UV illumination.

It is worthwhile to mention that, since the thickness of the fabricated Ga_2_O_3_ thin film is less than 500 nm in this work, the heterojunction formed between the Ga_2_O_3_ and the underlying GaN layer has also played a role on the performance of Ga_2_O_3_-based SBPDs. Specifically, due to the electron affinity and bandgap differences between Ga_2_O_3_ and GaN, their band alignment results in the formation of a type-II heterojunction [47], as illustrated in Figure 10b. At the interface, the conduction band offset creates a low potential barrier that facilitates the transfer of photogenerated electrons from Ga_2_O_3_ into the GaN layer. In contrast, the large valence band offset combined with the low hole mobility of GaN makes it difficult for holes in GaN to migrate into Ga_2_O_3_. Owing to its inherently higher electron mobility of GaN, GaN acts as a low-resistance transport channel for the injected electrons, thereby promoting more efficient carrier transport toward the electrodes [51], as shown in Figure 10c. Overall, the formation of Ga_2_O_3_ and GaN heterojunction is beneficial for improving the photocurrent of Ga_2_O_3_-based SBPDs. Finally, it is emphasized that, by optimizing the thermal oxidation process parameters for growing Ga_2_O_3_ thin films from GaN and by reducing the concentration of oxygen vacancies, the carrier-trapping effects of defect states can be effectively suppressed, thereby enhancing the photoresponse speed, stability, and overall performance of the photodetector.

## 4. Conclusions

In this work, Ga_2_O_3_ thin films were fabricated via PETO on the GaN/sapphire substrate, and the effect of varying O_2_-to-Ar ratios (2:1, 4:1, and 8:1) on the structural, morphological, and photoelectric properties of the resulting films is systematically studied. It is revealed that, at an optimal O_2_-to-Ar ratio of 4:1, the synthesized Ga_2_O_3_ thin film has the largest grain size of 31.4 nm, the fastest growth rate of 427.5 nm/hour, as well as the lowest oxygen vacancy concentration of 16.61%. Moreover, the growth mechanism of Ga_2_O_3_ thin films formed with PETO on the GaN/sapphire substrate is proposed. Subsequently, an MSM-structured photodetector based on the Ga_2_O_3_ thin film prepared at an optimal O_2_-to-Ar ratio of 4:1 was fabricated. The fabricated photodetector achieves a specific detectivity of $2.74\times 10^{13}$ Jones and a significant solar-blind/visible light rejection ratio of 116 at a bias voltage of 10 V. These findings offer an experimental foundation for optimizing the thermal oxidation process through atmospheric modulation and present a viable route for the cost-effective fabrication of Ga_2_O_3_ thin films for UV photodetector applications.
